# The Role of Red Blood Cell Distribution Width in the Severity and Prognosis of Community-Acquired Pneumonia

**DOI:** 10.1155/2021/8024024

**Published:** 2021-09-11

**Authors:** Qiang Ren, Hebing Liu, Ying Wang, Deyu Dai, Zhennan Tian, Guiwei Jiao, Xiaomin Liu

**Affiliations:** ^1^Respiratory Department, The First Affiliated Hospital of Harbin Medical University, Harbin, China; ^2^Tissue Microbiology Laboratory, Division of Cellular and Gene Therapies, Office of Tissues and Advanced Therapies, Center for Biologics Evaluation and Research, Food and Drug Administration, Silver Spring, MD 20993, USA

## Abstract

**Objectives:**

The objective of this study is to unravel the correlation between RDW and the severity and prognosis of CAP, as well as exploring RDW with the inflammatory markers white blood cells (WBC), C-reactive protein (CRP), and procalcitonin (PCT).

**Methods:**

According to the data characteristics, appropriate statistical methods were selected to analyze the relationship between RDW and the severity and prognosis of CAP patients and to determine whether RDW is associated with the inflammatory markers WBC, CRP, and PCT.

**Results:**

The results show that with the increase of PSI and CURB-65 values, the proportion of patients with RDW ≥ 12.987% is significantly higher than that of RDW < 12.987% (*P* < 0.01). When RDW is combined with PSI or CURB-65 to predict the 90-day mortality of CAP patients, the area under the receiver operating characteristic (ROC) curve increased prominently, and if RDW, PSI, and CURB-65 are combined, the area under the ROC curve is maximized.

**Conclusions:**

Our findings suggest that the higher RDW value is associated with short-term adverse outcomes in CAP patients. We also find that when RDW, PSI, and CURB-65 are combined, the best performance is achieved to predict CAP 90-day mortality risk.

## 1. Introduction

Community-acquired pneumonia (CAP) refers to the infectious pulmonary parenchymal inflammation acquired outside the hospital, and it is a worldwide public health problem. Studies have shown a mortality rate of less than 5% in outpatient CAP patients, more than 10% in hospitalized patients, and 30%if patients need to be admitted to the intensive care unit [1, 2]. Therefore, in the management of CAP, early identification of high-risk patients with poor prognosis is critical [3]. The widespread use of CAP severity assessment scales such as Pneumonia Severity Index (PSI) and CURB-65 has largely solved the problem of risk stratification, but it also has its own limitations. Recent studies have shown that certain biomarkers can make up for the shortcomings of the assessment scales, but they are expensive and not readily available [4]. The red blood cell distribution width (RDW) can be obtained immediately from blood routine report, which has the advantages such as simplicity and low cost, and is related to the severity and prognosis of CAP.

RDW is the variation coefficient of red blood cell volume in the circulating red blood cell system. This parameter is quantified as the standard deviation of red blood cell volume/mean red blood cell volume × 100%, which is expressed as a percentage [5]. RDW is rarely lower than the traditional reference range, while a higher than normal range reflects abnormal red blood cell homeostasis, normal red blood cell damage, and the presence of abnormal red blood cells, which may be attributed to (1) senescence of erythroid progenitor cells, followed by obstruction of erythroid proliferation and maturation disorders, resulted from shortening of telomere length [6, 7]; (2) imbalance of oxidation and antioxidation, increased production of reactive oxygen species, damaged substances including nucleic acid and protein, as well as interfered red blood cell homeostasis and survival due to oxidative stress [8–10]; and (3) inflammation impaired iron metabolism, interferes with the body's response to erythropoietin, resulting in a large number of immature red blood cells entering the circulatory system [11]. In addition, inflammation can directly affect the red blood cell survival rate, resulting in a mixed volume of red blood cells in the circulatory system [12].

The clinical application of RDW has long been limited to the combination of the average volume of red blood cells to identify the etiology and efficacy of anemia. However, several studies have shown that RDW values are closely associated with the cardiogenic and noncardiogenic mortality in people with coronary heart disease, acute and chronic heart failure, peripheral arterial disease, stroke, pulmonary thromboembolism, and pulmonary hypertension [13–18]. In addition, some studies have demonstrated that RDW values have predictive value for all-cause mortality in critically ill or ICU-hospitalized patients [19–22]. Salvagno et al. [23] have suggested that RDW values are considered to be strong and independent risk factors associated with death in the general population.

Recent studies have shown that RDW is also closely related to the severity and prognosis of infectious diseases [24]. RDW is significantly associated with short-term mortality in patients with CAP. The higher the RDW value, the greater the risk of short-term death [25]. A number of studies have shown that the scores of PSI and CURB-65 in CAP patients with higher RDW values also tend to be higher, and the higher the score level, the more severe the condition [26]. Many scholars have suggested that certain biomarkers can improve the evaluation performance of the above scoring system, such as procalcitonin, C-reactive protein, atrial natriuretic (ANP), and copeptin, which are currently widely studied. However, the high price of the above biomarkers has limited their clinical application [4]. Studies have shown that by including the RDW value as a severity assessment criterion, the prognostic evaluation performance of PSI and CURB-65 can be greatly improved [27]. RWD can be used as a marker to predict the severity of CAP patients without additional costs.

## 2. Materials and Methods

### 2.1. Design and Content

The study is a retrospective case analysis study of hospitalized patients with community-acquired pneumonia diagnosed in the Respiratory Department of Internal Medicine of the First Affiliated Hospital of Harbin Medical University from January 2016 to December 2018. All selected cases met the diagnostic criteria for community-acquired pneumonia. Exclusion criteria: (1) nosocomial acquired pneumonia; (2) pneumocystis pneumonia caused by HIV infection, chemotherapy, immunosuppressive agents, or long-term oral administration of large amounts of hormones; (3) active tuberculosis or suspected tuberculosis; (4) obstructive pneumonia caused by tumor or other reasons; (5) interstitial lung disease; (6) aspiration pneumonia; (7) patients with hematological diseases; (8) anemia patients, i.e., hemoglobin value: male < 120 g/L; female < 110 g/L. Ethical approval was obtained from the First Affiliated Hospital of the Harbin Medical University Ethics Committee.

### 2.2. Data Collection

CAP was defined as evidence of a pulmonary infiltrate on the chest radiograph and symptoms of lower respiratory infection, including cough, dyspnea, fever, and/or pleuritic chest pain, which were not acquired in a hospital or a nursing home. Data acquisition of incorporated CAP patients was carried out in the following aspects: (1) general information: gender, age, smoking history, drinking history, hospital stays; (2) pulmonary comorbidities: bronchial asthma, chronic obstructive pulmonary disease (COPD), old pulmonary tuberculosis, bronchiectasis, interstitial lung disease; (3) extrapulmonary disease: heart failure, liver disease, kidney disease, hypertension, diabetes, cerebrovascular disease, coronary heart disease, tumor, urinary tract infection, hyperthyroidism; (4) auxiliary examination: multiple pulmonary lobe infiltration, pleural effusion; (5) initial laboratory test indicators after admission: pH, PaO_2_, PaCO_2_, C-reactive protein (CRP), procalcitonin (PCT), white blood cells (WBC), platelets (PLT), hematocrit (HCT), red blood cell distribution width, hemoglobin (HGB), D-dimer (DDP), fibrinogen (FIB), albumin (ALB), blood urea nitrogen (BUN). Enrolled CAP patients were scored according to the CURB-65 and PSI rating scales, and the scores were accurately recorded. The 90-day mortality of patients with CAP was followed up and recorded.

### 2.3. Statistical Analysis

The statistical analysis of this study was completed with SPSS, version 20.0. The basic characteristic attributes of categorical variables are described by counting and composition percentage, and the basic characteristic attributes of quantitative variables are described by mean ± standard deviation. The Pearson chi-square test was used to compare the differences in the composition ratio between the two groups. The independent sample *T*-test was used to compare the differences between the groups. The Pearson correlation was used to analyze the correlation between the two groups. Finally, the logistic regression analysis was performed. The 90-day mortality risk factor was predicted, the ROC curve was plotted, the area under the curve was compared, and whether the RDW improved the evaluation performance of the PSI and CURB-65 scoring system was evaluated.

## 3. Results

### 3.1. Analysis of Clinical Data Characteristics of Different RDW Groups

A retrospective study is conducted on 3278 patients who met the screening criteria in our hospital from the collection period. The mean RDW of the sample is 12.987%, and all patients are divided into two groups according to the mean (Table 1). We find that the age and drinking history of CAP patients are statistically different between the two groups (*P* < 0.01). Higher RDW values are more likely to occur in elderly subjects and drinkers, while smoking has little effect on RDW values (*P* > 0.05).

In terms of comorbidity, the proportion of CAP patients with chronic obstructive pulmonary disease, cardiac insufficiency, kidney disease, hypertension, cerebrovascular disease, or coronary heart disease in the RDW ≥ 12.987% group is statistically higher than the proportion in the RDW < 12.987% group (*P* < 0.01); the proportion of CAP patients with old tuberculosis, interstitial lung disease, liver disease, diabetes, or oncology in the RDW ≥ 12.987% group is statistically higher than the proportion in the RDW < 12.987% group (*P* < 0.05); while there is no significant difference between the two groups in the proportion of CAP patients with bronchial asthma, bronchiectasis, urinary tract infection, and hyperthyroidism (*P* > 0.05).

Statistical analysis is also performed on the initial auxiliary examination results of patients admitted to the hospital. The proportion of CAP patients with pleural effusion or multiple pulmonary lobe infiltration is significantly higher in the RDW ≥12.987% group than in the RDW <12.987% group (*P* < 0.01). CAP patients with high RDW (in the RDW ≥12.987% group) tend to have low PaO_2_, high PaCO_2_, low HCT, low HGB, low ALB, or high BUN values (*P* < 0.01); while WBC, PLT, DDP, PCT, and CRP are not statistically different between the two groups (*P* > 0.05).

With the increase of PSI and CURB-65 scores, the proportion of patients with RDW ≥12.987% gradually turns from lower to higher than that of patients with RDW <12.987%, and the statistical difference is significant (*P* < 0.01). We speculated that CAP patients with higher RDW values may have higher PSI and CURB-65 scores.

### 3.2. Correlation Analysis between RDW and PSI, CURB-65, and Inflammatory Markers

To analyze the relationship between RDW in CAP patients and scoring systems (PSI, CURB-65), CAP inflammatory markers (WBC, PCT, and CRP), and other laboratory examination results, we used the Pearson correlation analysis for statistical processing (Table 2).

The results show that PSI and CURB-65 are significantly positively correlated with RDW, and the correlation coefficients are 0.207 and 0.192, respectively. The correlation between PSI and RDW is more significant than that of CURB-65. HGB, ALB, and HCT are negatively correlated with RDW, and their correlation coefficients are −0.115, −0.165, and −0.035, respectively; PaO_2_, BUN, and DDP have positive correlation with RDW, with correlation coefficients of 0.089, 0.067, and 0.055, respectively; no correlation is found between RDW and the CAP inflammatory markers WBC, PCT, or CRP.

### 3.3. Comparison of Clinical Data Characteristics between Survival and 90-Day Nonsurvival Group

To investigate whether RDW is associated with 90-day mortality of CAP patients, the cases are grouped by survival status (Table 3). In this study, 178 deaths occurred within 90 days, accounting for 5.43% of the total sample size. As shown in Table 3, the mean age of the deceased patient group is 74.421 ± 12.201 years old, and 55.075 ± 18.233 for the survival group. The statistical difference between the two groups is significant (*P* < 0.01). Smoking history is also statistically different between the survival group and the nonsurvival group (*P* < 0.01). Unlike age or smoking history, no significant difference is found in drinking history between the two groups (*P* > 0.05).

The distribution of CURB-65 is as follows: the survival group contains 3100 subjects in total, of which 1877 patients have the CURB-65 score of 0, 895 patients have the score of 1, 284 patients have the score of 2, 42 patients have the score of 3, and 2 patients have the score of 4, which account for 60.5%, 28.9%, 9.2%, 1.4%, and 0.1% of the total survival group, respectively; a total of 178 people are in the nonsurvival group, of which 12 patients have the CURB-65 score of 0, 55 patients with the score of 1, 64 patients with the score of 2, 40 patients with the score of 3, and 7 patients with the score of 4, accounting for 6.7%, 30.9%, 36.0%, 22.5%, and 3.9% of the nonsurvival group, respectively; no score of 5 is found in patients from both groups. From the distribution, we can see that as the CURB-65 score level increases, the proportion of patients in the nonsurvival group is gradually higher than that in the survival group, and the statistical difference is significant (*P* < 0.01).

The distribution of PSI is as follows: within the survival group, 1090 patients have a PSI grade of I, 902 patients have grade II PSI, 635 patients have grade III PSI, 408 patients have grade IV PSI, and 65 patients have grade V PSI, which represent 35.2%, 29.1%, 20.5%, 13.2%, and 2.1% of the total number of patients in the survival group, respectively; the number of patients with a PSI grade of I in the nonsurvival group is 3, the number of patients with the second grade is 5, the number of patients with grade III PSI is 10, the number of patients with grade IV PSI is 91, and the number of patients with grade V PSI is 69, accounting for 1.7%, 2.8%, 5.6%, 51.1%, and 38.8% of the total number of deceased subjects, respectively. The difference between the proportion of patients in the nonsurvival and the survival group is gradually smaller as the PSI grade increases. The proportion of patients in the nonsurvival group is higher than that in the survival group when comparing within PSI grade IV and V. Statistical analysis is performed on the above data, and the difference is statistically significant (*P* < 0.01).

Statistical analysis is performed on the difference of RDW between the survival and the nonsurvival group. As shown in Table 3, the mean RDW of the survival group is 12.918% ± 1.409%, and the mean value of RDW of the nonsurvival group is 14.184% ± 1.721%, which is significantly higher than that of the survival group (*P* < 0.01).

In addition, the statistical results show that the proportion of CAP patients with COPD in the nonsurvival group is significantly higher than that of the survival group, and the difference is statistically significant (*P* < 0.01). The initial WBC, PLT, HGB, DDP, ALB, and BUN values in the nonsurvival group are statistically significantly higher than those in the survival group (*P* < 0.01).

### 3.4. Predicting High-Risk Factors for 90-Day Mortality in CAP Patients Using Logistic Regression

To investigate the high-risk factors predicting 90-day mortality in CAP patients, logistic regression analysis is performed (Table 4), in which the interval of RDW is divided following relevant literature [26]. We observe that with the increase of the CURB-65 score, the OR value gradually increases. The risk of death for the CURB-65 score of 1, 2, 3, and 4 is 1.827, 2.221, 3.857, and 13.693 times higher than the risk of the CURB-65 score 0 group, respectively. As the PSI score level increases, the corresponding OR value also gradually increases. The risk of death for the PSI score level of II, III, IV, and V is 1.483, 3.050, 32.491, and 92.556 times higher than the death risk for the PSI level I group, respectively. Similarly, as the RDW level increases, the OR value also increases gradually. The risk of death in RDW value of 13.3%–14.1%, 14.1%–15.2%, and >15.2% group is 1.057, 3.644, and 5.519 times higher than that in the RDW <13.3% group, respectively. When the CURB-65 score is 3 or 4, the PSI level is IV or V, RDW value is 14.1%–15.2% or >15.2%, and the statistical difference is significant (*P* < 0.01).

### 3.5. Evaluating Whether RDW Can Improve PSI and CURB-65 Performance

**Figure 1:**
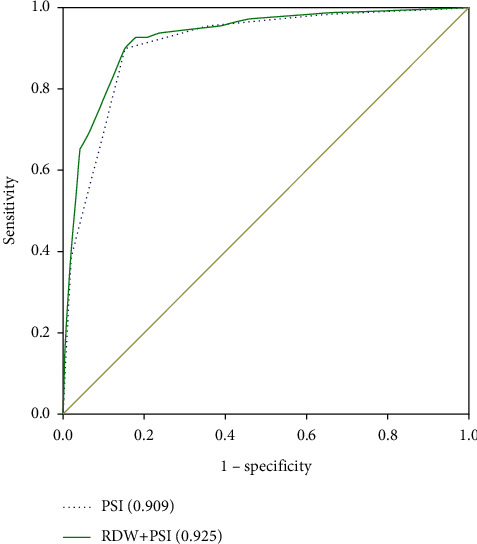
ROC curve of PSI alone and PSI combined with RDW to predict 90-day mortality.

**Figure 2:**
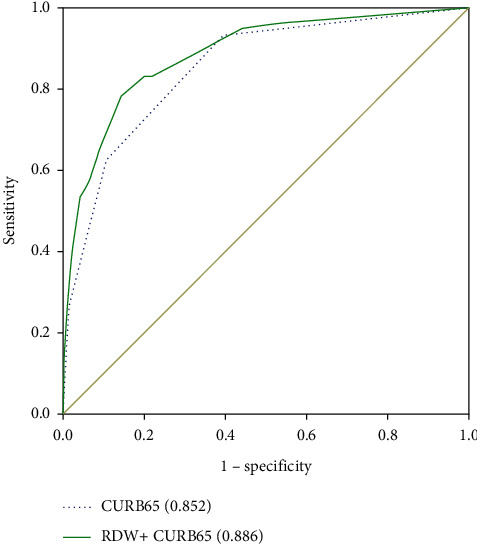
ROC curve of CURB-65 alone and CURB-65 combined with RDW to predict 90-day mortality.

**Figure 3:**
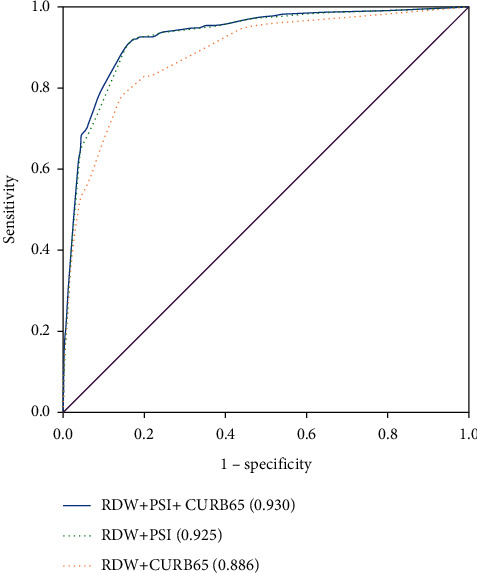
ROC curves of PSI and CURB-65 combined with RDW, respectively, and PSI with CURB-65 combined with RDW to predict 90-day mortality.

To evaluate whether RDW can improve the performance of the CAP severity scales PSI and CURB-65, the ROC curve is plotted. The area under the 90-day death curve using PSI and CURB-65 predictions is 0.909 (95%CI, 0.887–0.930) (Figure 1) and 0.852 (95%CI, 0.823–0.880) (Figure 2), respectively. After combining the PSI or CURB-65 scoring systems with RDW, the area under the ROC curve becomes 0.925 [95% CI, 0.905–0.945] (Figure 1) and 0.886 [95% CI, 0.859–0.912] (Figure 2), respectively, which significantly improved compared with the previous version (RDW vs. RDW + PSI, *P* < 0.001; RDW vs. RDW + CURB-65, *P* < 0.001). For RDW combining with PSI, sensitivity is 0.927, specificity is 0.829, PPV is 23.6%, and NPV is 99.5%. For RDW combining with CURB, sensitivity is 0.798, specificity is 0.848, PPV is 23.1%, and NPV is 98.6%. When PSI, CURB-65, and RDW are all combined, the area under the ROC curve increases to 0.930 [95% CI, 0.910–0.949] (Figure 3), and the improvement is the most obvious (RDW + PSI vs. RDW + CURB-65 + PSI, *P*=0.0103; RDW + CURB-65 vs. RDW + CURB-65 + PSI, *P* < 0.001). For RDW combining with PSI and CURB, sensitivity is 0.864, specificity is 0.904, PPV is 34.7%, and NPV is 99.1%.

## 4. Discussion

This study shows that patients with CAP who were admitted to our hospital with higher initial RDW values have higher PSI and CURB-65 scores, and the proportion of patients in the high-RDW group is gradually larger than that in the low-RDW group with the increase of PSI and CURB-65 scores. The severe patients are mainly distributed in the high-RDW group. Therefore, we believe that this parameter can reflect the severity of CAP to a certain extent, and there is a significant positive correlation between the two. In the cohort study, the RDW value is significantly higher in the nonsurvival group, suggesting that the higher RDW value is associated with short-term adverse outcomes in CAP patients. Some studies have confirmed that RDW change is an independent predictor of mortality in CAP patients [28].

Admittedly, the wide application of PSI and CURB-65 scoring systems in clinical practice has brought great benefits to the management of CAP. Clinicians can better identify high-risk and low-risk patients, so they can choose reasonable treatment, optimize the allocation of medical resources, patient treatment location, and the closeness of disease monitoring. However, these two assessment scales do have limitations. It is pointed out in the literature that they have only moderate sensitivity and specificity [29]. At the same time, one study [30] showed that RDW combined with WBC had a better sensitivity than CURB-65 scores in predicting ICU admission and/or mortality in CAP patients. Therefore, the ROC curve was drawn in this study. We found the predictive performance of PSI and CURB-65 scoring systems can be significantly improved by including RDW values, and when RDW, PSI, and CURB-65 are combined, the best performance is achieved to predict CAP 90-day mortality risk. Therefore, the introduction of RDW is a good complement to the deficiency of the pneumonia severity assessment scale and is obtained quickly without additional cost, which is different from PCT, copeptin, atrial natriuretic peptide, and other biomarkers.

Studies have shown that an elevated RDW value is associated with poor prognosis in a variety of human diseases, such as cardiovascular disease, venous thromboembolic disease, tumor, diabetes, liver and kidney disease, and chronic obstructive pulmonary disease [23]. Patients with CAP are often complicated by advanced disease and leading to poor prognosis. In this study, we find that the proportion of CAP patients with chronic obstructive pulmonary disease, heart failure, coronary heart disease, kidney disease, cerebrovascular disease, tumor, hypertension, or diabetes in the nonsurvival group is significantly higher than that in the survival group [31]. This result is consistent with our clinical experience. Interestingly, we also find when considering RDW values, the proportion of CAP patients with chronic obstructive pulmonary disease, heart failure, coronary heart disease, kidney disease, cerebrovascular disease, tumor, diabetes, or hypertension is significantly larger in the high-RDW group than that in the low-RDW group. The disease spectrum of the two RDW groups is very similar. We speculate that the greater the number of underlying diseases combined with hospitalized CAP patients, the greater the effect on RDW values and the higher the 90-day mortality rate. Higher RDW values may reflect the complexity of the combined diseases to some extent, suggesting poor prognosis.

The underlying cause of the association between elevated RDW values and CAP severity and poor prognosis is unclear. Erythropoietin, which regulates bone marrow production, red blood cell maturation, and survival, is previously considered to be one of the major determinants of RDW [32]. Its abnormal production or low reactivity of the body to erythropoietin will lead to a gradual increase in RDW values [33, 34]. CAP is a typical infectious disease, during which the inflammation stimulates the release of inflammatory factors, impairs the activity of erythropoietin, prevents red blood cell maturation, leads to the production of ineffective red blood cells, increases the unevenness of red blood cell sizes, and increases the RDW value [35]. Previous studies have shown a strong correlation between RDW and inflammatory markers, indicating that CRP and erythrocyte sedimentation rate (ESR) are high at high RDW values [36]. Therefore, this study specifically performed a correlation analysis between RDW and the inflammatory markers PCT, CRP, and WBC. However, the correlation between RDW and the inflammatory markers is not found, and the results are consistent with the research conducted by Lee et al. [26]. We speculate that this result may be due to (1) the limitations of the study; (2) the different changing phases between RDW and the three inflammatory markers under the action of inflammation; and (3) the WBC-, PCT-, and CRP-insensitive inflammatory mechanisms affecting the changes in RDW. The underlying cause is still unclear and needs further research to confirm. Oxidative stress is also very common in the pathophysiological process of CAP. For example, many hospitalized CAP patients often have decreased blood oxygen partial pressure. In severe cases, respiratory failure may occur, along with hypoxic tissue cells, resulting in an increase in reactive oxygen species, damage to living substances such as proteins and nucleic acids, red blood cell homeostasis, red blood cell heterogeneity, and increased RDW [35, 37]. In this study, we performed a correlation analysis between blood oxygen partial pressure and blood carbon dioxide partial pressure in RDW and blood gas analysis. The results show that RDW is negatively correlated with blood oxygen partial pressure and positively correlated with carbon dioxide partial pressure, which further proved the above point of view.

In addition, in our study, the levels of D-dimer, hematocrit, hemoglobin, albumin, and blood urea nitrogen are also significantly different in different RDW groups. We notice in the correlation analysis that RDW has the highest correlation with albumin, which is a commonly used parameter for laboratory tests of inpatients and a reliable prognostic marker for patients with severe infections [38, 39]. A low albumin level is associated with morbidity and mortality in a variety of diseases [40–44].

Although the sample size of the study is large enough, it is still a single-center retrospective study. The results are derived from the clinical information of CAP patients admitted to the First Affiliated Hospital of Harbin Medical University and might not be universally applicable. To promote the application, further multicenter prospective clinical studies are needed.

## Figures and Tables

**Table 1:** Data characteristic between the two groups.

	RDW < 12.987%	RDW ≥ 12.987%	*P* value
Age (years)	53.3 ± 18.1	59.9 ± 18.3	0.000
Hospitalization (days)	10.52 ± 4.66	10.41 ± 5.84	0.559
Smoker, *n* (%)	706 (37.8)	526 (37.7)	0.850
Drinker, *n* (%)	463 (24.8)	252 (17.9)	0.000
*Pulmonary comorbidities, n (%)*			
Bronchial asthma	79 (4.2)	42 (3.0)	0.062
COPD	148 (7.9)	164 (11.7)	0.000
Bronchiectasis	38 (2.0)	48 (3.1)	0.062
Old pulmonary tuberculosis	66 (3.5)	73 (4.2)	0.020
Interstitial lung disease	15 (0.8)	22 (1.6)	0.041

*Extrapulmonary diseases, n (%)*			
Heart failure	37 (2.0)	71 (5.0)	0.000
Liver disease	232 (12.4)	138 (11.3)	0.020
Renal disease	53 (2.8)	89 (6.3)	0.000
Hypertension	355 (19.0)	257 (25.0)	0.000
Diabetes	211 (11.3)	192 (13.6)	0.042
Cerebrovascular disease	195 (10.4)	270 (19.2)	0.000
Coronary heart disease	221 (11.8)	210 (14.9)	0.009
Tumor	54 (2.9)	60 (4.3)	0.033
Urinary tract infection	115 (6.1)	104 (7.4)	0.160
Hyperthyroidism	12 (0.6)	11 (0.8)	0.633

*Auxiliary examination*			
Pleural effusion, *n* (%)	369 (19.7)	350 (24.9)	0.000
Multiple pulmonary lobe infiltration, *n* (%)	920 (49.3)	789 (56.1)	0.000
pH	7.444 ± 0.049	7.4956 ± 2.000	0.424
PaO_2_ (mmHg)	70.476 ± 16.386	67.695 ± 17.744	0.000
PaCO_2_ (mmHg)	36.654 ± 6.830	37.770 ± 9.189	0.002
WBC (10^9^/L)	8.920 ± 15.004	9.247 ± 5.977	0.391
PLT (10^9^/L)	232.037 ± 85.554	237.232 ± 94.415	0.100
HCT (%)	40.843 ± 13.331	39.739 ± 5.732	0.001
HGB (g/L)	138.058 ± 14.211	134.378 ± 15.528	0.000
DDP (*μ*g/L)	1.619 ± 7.103	2.000 ± 4.719	0.079
FIB (g/L)	4.442 ± 1.755	4.280 ± 1.627	0.013
ALB (g/L)	37.762 ± 5.460	36.307 ± 5.975	0.000
BUN (mmol/L)	5.283 ± 11.210	6.105 ± 4.191	0.009
PCT (ng/L)	0.828 ± 4.460	1.166 ± 5.748	0.194
CRP (mg/L)	63.211 ± 81.097	68.749 ± 83.384	0.358

*PSI, n (%)*			0.000
I	729 (39.0)	366 (26.0)	
II	535 (28.6)	370 (26.3)	
III	339 (18.1)	305 (21.7)	
IV	227 (12.1)	272 (19.3)	
V	41 (2.2)	94 (6.7)	

*CURB-65, n (%)*			0.000
0	1230 (65.8)	659 (46.8)	
1	465 (24.9)	485 (34.4)	
2	151 (8.1)	197 (14.0)	
3	23 (1.2)	59 (4.2)	
4	1 (0.1)	8 (0.6)	
5	0	0	

**Table 2:** Correlation of RDW and PSI, CURB-65, and inflammatory biomarkers.

	Coefficient of correlation (*p*)	
	RDW	*P* value
PSI	0.207^*∗∗*^	0.000
CURB-65	0.192^*∗∗*^	0.000
HGB	−0.115^*∗∗*^	0.000
ALB	−0.165^*∗∗*^	0.000
BUN	0.067^*∗∗*^	0.000
DDP	0.055^*∗∗*^	0.002
HCT	−0.035^*∗*^	0.042
PaO_2_	0.089^*∗∗*^	0.000
WBC	0.020	0.242
PCT	0.041	0.108
CRP	0.026	0.473

^*∗∗*^represents that the correlation is significant at a significance level of 0.01; ^*∗*^represents that the correlation is significant at a significance level of 0.05.

**Table 3:** Data characteristic between survival group and nonsurvival group.

	Survival group	Nonsurvival group	*P* value
Age (years)	55.1 ± 18.2	74.4 ± 12.2	0.000
Smoker, *n* (%)	1143 (37.0)	89 (50.0)	0.000
Drinker, *n* (%)	674 (21.8)	41 (23.0)	0.693

*Pulmonary comorbidities, n (%)*			
Bronchial asthma	116 (3.7)	5 (2.8)	0.021
COPD	282 (9.1)	30 (16.9)	0.001
Bronchiectasis	77 (2.5)	4 (2.2)	0.843
Old pulmonary tuberculosis	127 (4.1)	12 (6.7)	0.089
Interstitial lung disease	34 (1.1)	3 (1.7)	0.470

*Extrapulmonary diseases, n (%)*			
Heart failure	79 (2.5)	29 (16.3)	0.000
Liver disease	346 (11.2)	24 (13.5)	0.341
Renal disease	109 (3.5)	33 (18.5)	0.000
Hypertension	653 (21.1)	59 (33.1)	0.000
Diabetes	361 (23.6)	42 (11.6)	0.000
Cerebrovascular disease	381 (12.3)	84 (47.0)	0.000
Coronary heart disease	393 (12.7)	38 (21.3)	0.001
Tumor	96 (3.1)	18 (10.1)	0.000
Urinary tract infection	208 (6.7)	11 (6.2)	0.783
Hyperthyroidism	22 (0.7)	1 (0.6)	0.882

*Auxiliary examination*			
Pleural effusion, *n* (%)	637 (20.6)	82 (46.1)	0.000
Multiple pulmonary lobe infiltration, *n* (%)	1709 (52.2)	125 (71.0)	0.000
pH	7.423 ± 0.104	7.471 ± 1.414	0.190
PaO_2_ (mmHg)	69.955 ± 16.498	59.487 ± 20.976	0.000
PaCO_2_ (mmHg)	37.156 ± 7.398	37.273 ± 14.482	0.750
WBC (10^9^/L)	8.904 ± 12.208	11.782 ± 6.657	0.000
PLT (10^9^/L)	235.509 ± 88.709	212.662 ± 88.709	0.001
RDW (%)	12.918 ± 1.409	14.184 ± 1.721	0.000
HGB (g/L)	136.648 ± 14.581	133.529 ± 19.462	0.007
DDP (*μ*g/L)	1.586 ± 5.818	5.014 ± 10.109	0.000
FIB (g/L)	4.507 ± 1.759	4.364 ± 1.699	0.307
ALB (g/L)	37.517 ± 5.472	29.516 ± 5.533	0.000
BUN (mmol/L)	5.330 ± 8.905	10.918 ± 7.502	0.000
PCT (ng/L)	0.7878 ± 4.211	5.3863 ± 13.970	0.000
CRP (mg/L)	61.523 ± 78.174	142.611 ± 112.961	0.000

*CURB-65, n (%)*			0.000
0	1877 (60.5)	12 (6.7)	
1	895 (28.9)	55 (30.9)	
2	284 (9.2)	64 (36.0)	
3	42 (1.4)	40 (22.5)	
4	2 (0.1)	7 (3.9)	
5	0	0	

*PSI, n (%)*			0.000
I	1090 (35.2)	3 (1.7)	
II	902 (29.1)	5 (2.8)	
III	635 (20.5)	10 (5.6)	
IV	408 (13.2)	91 (51.1)	
V	65 (2.1)	69 (38.8)	

**Table 4:** Logistic regression result for predicting 90-d death.

Variable	OR	95%CI	*P* value
CURB-65 0	—		—
CURB-65 1	1.827	0.847–3.941	0.125
CURB-65 2	2.221	0.985–4.956	0.054
CURB-65 3	3.857	1.438–10.083	0.007
CURB-65 4	13.693	2.107–88.978	0.006
PSI grade I	—		—
PSI grade II	1.483	0.340–6.466	0.600
PSI grade III	3.050	0.744–12.503	0.121
PSI grade IV	32.491	8.571–123.166	0.000
PSI grade V	92.556	22.344–383.476	0.000
RDW < 13.3%	—		—
RDW 13.3%–14.1%	1.057	0.633–1.768	0.083
RDW 14.1%–15.2%	3.644	2.196–6.115	0.000
RDW > 15.2%	5.519	3.287–9.267	0.000

OR: odds ratio.

## Data Availability

All data included in this study are available upon request by contact with the corresponding author.
